# Parathyroidectomy Versus Cinacalcet for the Treatment of Secondary Hyperparathyroidism in Hemodialysis Patients

**DOI:** 10.1007/s00268-022-06439-7

**Published:** 2022-01-12

**Authors:** Luis Alvarado, Nishtha Sharma, Roxann Lerma, Alok Dwivedi, Adeel Ahmad, Aimee Hechanova, Fernanda Payan-Schober, Azikiwe Nwosu, Eyas Alkhalili

**Affiliations:** Texas Tech Health Sciences Center El Paso, 2000B Woodrow Bean Transmountain Rd., B323, El Paso, TX 79911 USA

## Abstract

**Background:**

Secondary hyperparathyroidism in patients with end stage renal disease on dialysis is associated with bone pain and fractures in addition to cardiovascular morbidity. Cinacalcet is the most commonly used drug to treat such patients, but it has never been compared to surgery. The goal of this study is to compare the long-term outcomes and survival between cinacalcet and parathyroidectomy in the treatment of secondary hyperparathyroidism in hemodialysis patients.

**Methods:**

Adult patients on hemodialysis who were treated with cinacalcet or parathyroidectomy in the United States Renal Data System were included. Patients treated with surgery (*n* = 2023) were compared using 1:1 propensity score matching ratio to a cohort of patients treated with cinacalcet. A Cox regression analysis was conducted to compare the overall mortality.

**Results:**

The propensity score matching successfully created two groups with similar demographics. Patients in the surgery group had a higher mean peak PTH level prior to therapy (2066.8 vs 1425.4, *P* < 0.001). No difference was observed in the development of new-onset coronary artery disease (7.7% vs 7.9%, *P* = 0.8) or cerebrovascular disease (7% vs 6.7%, *P* = 0.8). Surgical patients had a higher rate of pathologic fractures (27.8% vs 24.9%, *P* = 0.04). Survival analysis showed that patients undergoing surgery had a better 5-year survival (65.6% vs 57.8%) and were less likely to die within the study period (HR 0.77, 95% CI 0.7–0.85, *P* < 0.0001).

**Conclusions:**

Patients on dialysis undergoing parathyroidectomy for the treatment of secondary hyperparathyroidism have a better overall survival than those treated with cinacalcet.

## Background

Secondary hyperparathyroidism in patients with end stage renal disease (ESRD) on dialysis is associated with bone pain and fractures in addition to accelerated vascular calcification. It has been shown that elevated levels of phosphorus, parathyroid hormone (PTH), calcium, alkaline phosphatase, and fibroblast growth factor 23 are associated with cardiovascular morbidity and mortality [1–4].

Cinacalcet (Sensipar/Mimpara, Amgen) is a calcimimetic that activates the calcium-sensing receptor (CSR) on the parathyrocytes that is approved for the treatment of secondary hyperparathyroidism after showing effectiveness in reducing the levels of PTH levels as well as calcium and phosphorus levels in randomized controlled trials [5, 6]. The Evaluation of Cinacalcet Therapy to Lower Cardiovascular Events (EVOLVE) trial, however, showed that Cinacalcet did not reduce the risk of death or major cardiovascular morbidity in patients with secondary hyperparathyroidism who are undergoing dialysis [7].

The Kidney Disease: Improving Global Outcomes (KDIGO) CKD-MBD Update Work Group recommends calcimimetics such as Cinacalcet, Calcitriol, Vitamin D analogs, or a combination of calcimimetics with calcitriol or vitamin D analogs in patients requiring PTH-lowering therapy. The guidelines also recommend parathyroidectomy in patients with severe hyperparathyroidism who fail to respond to medical therapy [4].

Parathyroidectomy for secondary hyperparathyroidism in patients with ESRD has been shown to have a higher complication and death rate compared to parathyroidectomy when performed for other indications [8]. As such, surgery has been reserved for patients with refractory disease that is otherwise uncontrolled with medical therapy. There has been a single prospective randomized study comparing parathyroidectomy with Cinacalcet in treating hyperparathyroidism, but it was performed in patients with persistent hyperparathyroidism after kidney transplantation (tertiary hyperparathyroidism). The study showed that parathyroidectomy was superior to Cinacalcet in achieving normocalcemia and improving bone mass density (BMD) [9].

The aim of this study is to compare the long-term outcomes and survival between Cinacalcet and parathyroidectomy in patients with secondary hyperparathyroidism receiving hemodialysis using The United States Renal Data System (USRDS).

## Methods

### Data source and study population

The USRDS is a national registry of ESRD patients which includes data from hundreds of participating hospitals across the US. www.usrds.org. The following Standard Analysis Files (SAF) from the USRDS data were used and merged to conduct the analysis: Core, Hospital, CROWNWeb clinical data, Institutional Claims, Medicare Claims, Physician/Supplier claims, and Part D.

We included patients on hemodialysis aged ≥18 years treated between January 1, 2012, and December 31, 2014, with a PTH level ≥1000 pg/mL. The study population was divided into two groups. The surgery group was identified using the current procedural terminology (CPT) for parathyroidectomy (60500), while the second group included patients who were treated with Cinacalcet during the study time-frame.

### Patients characteristics

Patients’ demographics and clinical characteristics were compared including age, sex, race, body mass index (BMI), and comorbid conditions. These were defined using ICD-9 and ICD-10 codes. We also included laboratory values such as albumin, hemoglobin, calcium, phosphorus, and PTH levels.

### Defining outcomes

The outcomes of interest were death during the study period, overall survival, hospitalizations after initiating treatment (surgery or medical) that are related to cardiovascular or bone disease, and any procedures or surgeries performed to treat those conditions.

### Statistical analysis

Due to the inequality in clinical variables between the two groups, a propensity match score was conducted. The group treated with surgery (S) was compared using 1:1 propensity score matching ratio to a cohort of patients treated with Cinacalcet (C). The groups were matched for age, sex, race, preoperative albumin, and hemoglobin levels, existing comorbidities (including diabetes, COPD, and cancer), and receiving a kidney transplant.

Qualitative variables were reported using frequencies and percentages, while quantitative variables were reported using means and standard deviations. The association between the variables was analyzed using an unpaired *t*-test, Chi-square test, and Fisher’s exact test. A relative risk regression was conducted to assess the differences between the treatment groups.

A Cox regression analysis was also conducted to compare the overall mortality after surgery or medication. A Kaplan–Meier Curve was generated to show the overall probability of death after receiving either treatment. Hazard ratio (HR), 95% confidence interval (C.I.), and *P* values were used to describe the models. *P* values were considered statistically significant when <0.05. All data analysis and management was conducted using Stata 15 (StataCorp. 2017 LLC, College Station, TX, USA) and SAS Version 9 (SAS Institute Inc., Cary, NC, USA).

## Results

There were 2023 patients in each group. The propensity score matching successfully created two groups with similar demographics (Table 1). Patients in the surgery group had a higher mean peak PTH level prior to therapy (S 2066.8 vs C 1425.4 pg/mL, *P* < 0.001). The 30-day postoperative mortality in the surgery group was 3.7%. The post-intervention nadir PTH was lower in the surgery group (S 67.8 vs 516.3 pg/mL, *P* < 0.001). Seventeen percent of patients who underwent surgery received treatment with Cinacalcet during the study period indicating surgical failure or disease recurrence.

**Table 1:** Demographic variables

	Cinacalcet *N* = 2023	Parathyroidectomy *N* = 2023	*P* value
Age at time of treatment, mean (SD)	49.0 (13.8)	49.5 (13.0)	0.21
BMI, mean (SD)	31.4 (9.1)	31.4 (8.7)	0.96
Race			0.30
White	741 (36.6%)	781 (38.6%)	
Black	1192 (58.9%)	1145 (56.6%)	
Native American	19 (0.9%)	21 (1.0%)	
Asian	36 (1.8%)	30 (1.5%)	
Pacific Islander	19 (0.9%)	16 (0.8%)	
Other	14 (0.7%)	25 (1.2%)	
Don't know	2 (0.1%)	5 (0.2%)	
Sex			0.71
Female	979 (48.4%)	992 (49.0%)	
Male	1044 (51.6%)	1031 (51.0%)	
Albumin (g/dL), median (IQR)	4.0 (3.8, 4.2)	4.0 (3.8, 4.2)	0.72
Hemoglobin (g/dL), mean (SD)	10.5 (2.1)	10.5 (2.2)	0.78
Calcium corrected (md/dL), mean (SD)	9.2 (0.8)	9.2 (0.9)	0.095
Phosphorus (mg/dL), mean (SD)	6.5 (1.9)	6.7 (2.0)	< 0.001
Peak PTH (pg/dL), median (IQR)	1425.4 (1174.3, 1865.7)	2066.8 (1485.2, 2965.3)	< 0.001
Nadir PTH (pg/dL) after treatment, median (IQR)	516.3 (240.5, 1020.9)	67.8 (12.4, 250.1)	< .0001
Diabetes	475 (23.5%)	481 (23.8%)	0.85
Kidney transplant	673 (33.3%)	677 (33.5%)	0.92
COPD	45 (2.2%)	47 (2.3%)	0.92
Malignant neoplasm, cancer	38 (1.9%)	37 (1.8%)	1.00
Alcohol dependence	22 (1.1%)	22 (1.1%)	1.00

No difference was observed in the development of new-onset coronary artery disease (S 7.7% vs C 7.9%, *P* = 0.8) or cerebrovascular disease (S 7% vs C 6.7%, *P* = 0.8). Surgical patients had a higher rate of calcific uremic arteriolopathy (calciphylaxis) (S 2.3% vs C 1.1%, *P* = 0.007) and higher rate of pathologic fractures (S 27.8% vs C 24.9%, *P* = 0.035) (Table 2).

**Table 2:** Clinical outcomes

	Cinacalcet *N* = 2023	Parathyroidectomy *N* = 2023	*P* value
Osteoporosis	59 (2.9%)	55 (2.7%)	0.78
Pathologic fractures	503 (24.9%)	563 (27.8%)	0.04
Calciphylaxis	23 (1.1%)	46 (2.3%)	0.007
Skin/soft tissue debridement	141 (7.0%)	135 (6.7%)	0.76
Coronary artery disease	156 (7.7%)	158 (7.8%)	0.95
Cerebrovascular disease	136 (6.7%)	141 (7.0%)	0.8
Peripheral vascular disease	261 (12.9%)	255 (12.6%)	0.81
Cardiac dysrhythmia	4 (0.2%)	7 (0.4%)	0.55
Pericarditis	2 (0.1%)	2 (0.1%)	1.00
Congestive heart failure	324 (16.0%)	310 (15.3%)	0.57
Death within study period	856 (42.3%)	725 (35.8%)	<0.001
Median survival in months, median (IQR)	18 (9.2, 35.9)	28 (13.7, 42.4)	<0.001
*Cause of death*			0.049
Coronary artery disease	32 (4.1%)	31 (4.7%)	
Cerebrovascular disease	16 (2.0%)	24 (3.6%)	
Sepsis	86 (11.0%)	66 (10.0%)	
Bleeding/cancer/other	118 (15.0%)	121 (18.3%)	
Uremia	51 (6.5%)	57 (8.6%)	
Undocumented	482 (61.4%)	362 (54.8%)	

*IQR* interquartile range

**Fig. 1:**
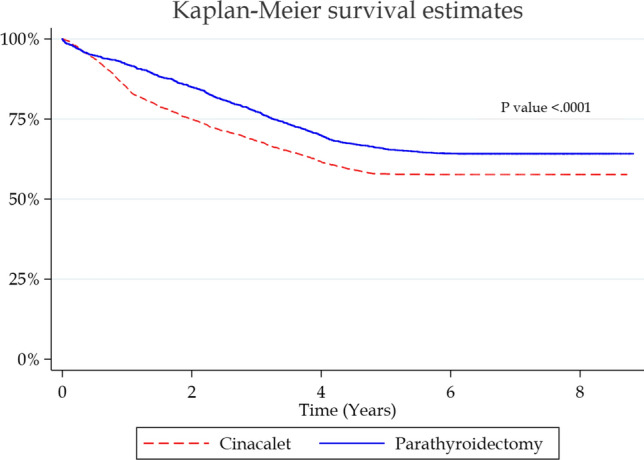
Kaplan–Meier survival curve comparing treatment groups in years (parathyroidectomy: solid blue; cinacalcet: dashed red)

Survival analysis showed that patients undergoing surgery had a better 5-year survival (S 65.7% vs C 57.9%, *P* < 0.0001) (Fig. 1). Cox regression mortality analysis showed that patients in the surgical group were less likely to die within the study period (HR 0.77, 95% CI 0.7–0.85, *P* < 0.0001). When we performed Cox regression analysis with PTH included and excluded from the propensity matching, PTH appeared to have no significant effect on the model and it resulted in similar findings (Table 3).

**Table 3:** Cox regression analysis models including and excluding PTH from the propensity matching

	Restricted time ≤6.2 years		
	HR	95% CI	*P* value
Model 1			
Parathyroidectomy	0.563	0.51, 0.63	<.0001
(Ref: Cinacalcet)			
Model 2			
Parathyroidectomy	0.58	0.52, 0.64	<.0001
(Ref: Cinacalcet)			
Model 3			
Parathyroidectomy	0.55	0.49, 0.61	<.0001
(Ref: Cinacalcet)			

Model 1: PTH serum was included in the propensity matching

Model 2: PTH serum was excluded from the propensity matching

Model 3: PTH serum was excluded from the propensity matching and adjusted for within the cox regression

The cause of death was not known or recorded simply as “cardiac arrest” in 57% of patients and as such we were not able to conduct a meaningful analysis comparing the cause of death in each group.

Multivariable hazards regression analysis showed that patients in the surgical group were more likely to develop pathologic fractures (HR 1.12, 95% CI 1.01–1.24, *P* = 0.03), but no difference in the development of cardiovascular disease was observed (Table 4). We performed a sub-group analysis on the patients who underwent kidney transplant. There was no difference in the rate of pathologic fractures, but patients who underwent surgery had a lower risk of soft-tissue debridement and congestive heart failure (Table 5). Surgical patients who underwent transplant were like likely to die than those were treated with cinacalcet and underwent transplant (HR 0.47, 95% CI 0.396–0.564, *P* < 0.0001) (Table 6).

**Table 4:** Cox proportional hazards model analysis (Ref: Cinacalcet)

Outcome	HR	95% CI		*P* value
Pathologic fractures	1.12	1.01	1.24	0.03
Congestive heart failure	0.95	0.83	1.10	0.55
Coronary artery disease	1.01	0.82	1.25	0.91
Cerebrovascular disease	1.03	0.83	1.30	0.76
Peripheral vascular disease	0.97	0.83	1.15	0.77

*HR* hazard ratio

**Table 5:** Relative risk model analysis of outcomes in the subgroup of patients who underwent kidney transplant (Ref: Cinacalcet)

Outcome	RR	95% CI		*P* value
Skin/soft tissue debridement	0.64	0.42	0.98	0.041
PAD	0.83	0.62	1.11	0.207
Congestive heart failure	0.77	0.61	0.97	0.032
Atherosclerotic heart disease	0.78	0.53	1.17	0.244
Coronary artery disease	0.82	0.57	1.17	0.287
Cerebrovascular disease	0.58	0.32	1.04	0.069
Peripheral vascular disease	0.73	0.44	1.2	0.217
Any cardiovascular intervention	0.97	0.74	1.26	0.817
Pathologic fractures	1.03	0.91	1.16	0.613

*RR* relative risk

**Table 6:** Cox regression analysis with risk of death in the outcomes in the subgroup of patients who underwent kidney transplant (Ref: Cinacalcet)

	HR	95% CI	*P* value
*Model 1*			
Parathyroidectomy	0.474	0.398, 0.564	<.0001
(Ref: Cinacalcet)			
*Model 2*			
Parathyroidectomy	0.474	0.396, 0.561	<.0001
(Ref: Cinacalcet)			

Model 1: PTH serum was excluded from the model

Model 2: PTH serum was adjusted for within the cox regression

## Discussion

In this large database study, we showed that hemodialysis patients with severe secondary hyperparathyroidism have better survival when treated by parathyroidectomy than those treated with Cinacalcet despite having more severe disease. To our knowledge, this is the first study to compare the outcomes between these treatment modalities.

The rate of parathyroidectomy to treat secondary hyperparathyroidism has decreased in recent years, mostly due to the expansion of medical therapeutic options to treat the disease [10, 11]. Surgery is associated with a low risk of recurrent disease, improved quality of life, lower risk of tertiary hyperparathyroidism, and decreased risk of graft failure in transplant recipients [12–15]. In 2004, Kestenbaum et al. have reported improved survival following parathyroidectomy in patients with secondary hyperparathyroidism when compared to those who did not undergo surgery [16]. Our study shows that the survival advantage of surgery over medical therapy persists more than a decade later and surpasses that of Cinacalcet. Parathyroidectomy in ESRD patients, however, is associated with significant perioperative risks including a two-percent 30-day postoperative mortality reported in the literature and 3.7% mortality risk in our study [8]. Due to these risks, clinicians follow the KDIGO guidelines and only refer patients with severe refractory disease for surgery [4].

While Cinacalcet has been shown to lower PTH levels and improve calcium-phosphorus homeostasis, it failed to reduce the risk of death or major cardiovascular events as shown in the EVOLVE trial [6, 7]. After the EVOLVE trial showed that Cinacalcet decreased the rate of parathyroidectomy, the rate of parathyroidectomies in the USA decreased between 2004 and 2005 with Cinacalcet made available; however, the rate of surgery rose again shortly after. The suggested rationale is that the severity of the side effects caused the doses used in clinical practice to be much lower than those used in the trial [17]. The other drawback to Cinacalcet use is the high cost [18].

Despite these drawbacks, Cinacalcet remains a reasonable treatment option for secondary hyperparathyroidism. It is important to point out that all the studies thus far have compared Cinacalcet to other medical treatments but not to surgery. The only study to compare surgery to Cinacalcet was in patients with hypercalcemia in transplant recipients (Tertiary hyperparathyroidism), and it showed that surgery was superior in correcting hypercalcemia [9].

The low nadir PTH in our parathyroidectomy cohort is likely explained by aggressive resection. Other studies have shown similar findings of a suppressed nadir PTH following surgery [19]. The consequence of such surgical approach during parathyroidectomy is the over suppression of PTH and the development of adynamic bone disease which predisposes to skeletal pain and fractures (particularly when PTH levels drop below 100 pg/mL) [20, 21]. This is a potential explanation of the slightly higher incidence of pathologic fractures in our surgical cohort. Another explanation would be the higher level of peak PTH in the surgical cohort.

Our study has a few limitations. First is its retrospective nature and the lack of randomization which made for important differences in baseline clinical characteristics between the two groups. Selection bias would mean that healthier patients are more likely to be offered surgery. We were able to create similar cohorts by the use of propensity-match scoring to minimize the effects of these factors. The use of a large outcomes database makes it difficult to track and extract certain relevant data as was apparent in the lack of exact cause of death in most patients, making a meaningful direct cause of mortality analysis difficult to conduct.

In conclusion, this large database study, the first of its kind, shows that parathyroidectomy is associated with an overall better survival than Cinacalcet in patients with secondary hyperparathyroidism on hemodialysis. Surgery should be strongly considered particularly in patients with PTH levels higher than 1000 pg/dL. While the conduct of a randomized prospective study comparing the two treatment modalities is feasible, it is certainly an enormous undertaking and unlikely to be performed soon. Because of this, the results of this analysis are an invaluable asset to help guide decision making for clinicians caring for these complex patients.

## Abbreviations

ESRD: End-stage renal disease
PTH: Parathyroid hormone
KDIGO: The Kidney Disease: Improving Global Outcomes
BMD: Body mass density
USRDS: The United States Renal Data System
BMI: Body mass index
COPD: Chronic obstructive pulmonary disease
HR: Hazard ratio
RR: Relative risk
CI: Confidence interval
